# Selenium Deficiency Leads to Inflammation, Autophagy, Endoplasmic Reticulum Stress, Apoptosis and Contraction Abnormalities *via* Affecting Intestinal Flora in Intestinal Smooth Muscle of Mice

**DOI:** 10.3389/fimmu.2022.947655

**Published:** 2022-07-06

**Authors:** Fuhan Wang, Ni Sun, Hanqin Zeng, Yuan Gao, Naisheng Zhang, Wenlong Zhang

**Affiliations:** College of Veterinary Medicine, Jilin University, Changchun, China

**Keywords:** intestinal flora, inflammatory, autophagy, tight junction, apoptosis, ERS, smooth muscle contraction, selenium

## Abstract

Selenium (Se) is a micronutrient that plays a predominant role in various physiological processes in humans and animals. Long-term lack of Se will lead to many metabolic diseases. Studies have found that chronic Se deficiency can cause chronic diarrhea. The gut flora is closely related to the health of the body. Changes in environmental factors can cause changes in the intestinal flora. Our study found that Se deficiency can disrupt intestinal flora. Through 16s high-throughput sequencing analysis of small intestinal contents of mice, we found that compared with CSe group, the abundance of Lactobacillus, Bifidobacterium, and Ileibacterium in the low selenium group was significantly increased, while Romboutsia abundance was significantly decreased. Histological analysis showed that compared with CSe group, the small intestine tissues of the LSe group had obvious pathological changes. We examined mRNA expression levels in the small intestine associated with inflammation, autophagy, endoplasmic reticulum stress, apoptosis, tight junctions, and smooth muscle contraction. The mRNA levels of *NF-κB*, *IκB*, *p38*, *IL-1β*, *TNF-α*, *Beclin*, *ATG7*, *ATG5*, *LC3α*, *BaK*, *Pum*, *Caspase-3*, *RIP1*, *RIPK3*, *PERK*, *IRE1*, *elF2α*, *GRP78*, *CHOP2*, *ZO-1*, *ZO-2*, *Occludin*, *E-cadherin*, *CaM*, *MLC*, *MLCK*, *Rho*, and *RhoA* in the LSe group were significantly increased. The mRNA levels of *IL-10*, *p62 BcL-2* and *BcL-w* were significantly decreased in the LSe group compared with the CSe group. These results suggest that changes in the abundance of Lactobacillus, bifidobacterium, ileum, and Romboutsia may be associated with cellular inflammation, autophagy, endoplasmic reticulum stress, apoptosis, tight junction, and abnormal smooth muscle contraction. Intestinal flora may play an important role in chronic diarrhea caused by selenium deficiency.

## 1 Introduction

Selenium (Se) is a micronutrient that plays a vital role in various physiological processes in humans and animals. Lots of previous research have confirmed the biological effects of Se. Se has antioxidant properties, anti-cancer, enhances immunity, and other efficacy (1–3). Humans and animals need to continuously obtain nutrients from the external world to maintain normal physiological processes. Se is one of them. The body must maintain a balanced Se concentration (4). Long-term Se deficiency can lead to many diseases, such as cancer, liver disease, cardiovascular disease, pancreatic disease, cataracts, diabetes, and other diseases (5–13). Some studies have found that many animals with selenium deficiency develop diarrhea symptoms (14–16). Previous studies have shown that environmental factors can cause changes in the intestinal flora in the body (17–19). Does Se deficiency cause changes in intestinal flora? Is it because of this change that diarrhea develops? What is the mechanism?

The various bacteria that live in the gut are called the gut microbiome, and there are about 10 trillion of them. It contains 100 to 1000 bacterial species and 50 phyla. The gut microbiota is called the second gene (20). With the continuous progress of genome sequencing, culture omics, and other technologies, people are increasingly exploring the structure and function of intestinal flora (21). The regular operation of the intestinal tract must keep the dynamic and relative proportional balance among various microorganisms in the intestinal tract (22). According to their different roles in the gut, they are classified as beneficial, opportunistic, and pathogenic bacteria (23). Intestinal flora is a symbiotic relationship with human beings and plays a very important role in human life and health (24). They play an important role in the immune system, metabolic system, nervous system, and so on (25–27). When the composition of the gut microbiome changes, its function may change, which in turn affects the health of animals. This condition, called intestinal microbiome disorder, can cause diseases of the digestive, nervous, respiratory, and vascular systems (28–31). In recent years, intestinal flora has become a new hot research field. The interaction between intestinal flora and Se in food mainly focuses on the composition of symbiotic flora, the regulation of the metabolic process, and the function of the intestinal mucosal barrier (32, 33). Exploring the relationship between intestinal flora and dietary selenium can provide a theoretical basis for the treatment of intestinal diseases.

Diarrhea is associated with intestinal inflammation, disruption of the intestinal mucosal barrier, and abnormal contraction of the intestinal smooth muscle (34–37). Previous studies have found that bacterial enteritis is associated with disruption of intestinal barrier function (38). Intestinal inflammatory diseases are characterized by intestinal inflammation and the damage of the intestinal mucosa to varying degrees. Widespread epithelial cell death is one of them (39, 40). The release of inflammatory factors can induce apoptosis, necrosis, and autophagy of intestinal tissue cells. Under the action of various stimulating factors, such as infection and oxidative stress, the endoplasmic reticulum will become dysfunctional, leading to endoplasmic reticulum stresses (ERS) (41). Studies have shown that the intestinal biodiversity of ulcerative colitis (UC) and Crohn’s disease patients decreased, and the dominant flora and opportunistic pathogens changed (42, 43). Abnormal contraction of intestinal smooth muscle can cause diarrhea with intestinal motility disorder (44).

An intact intestinal mucosal barrier is essential for maintaining the host’s physiological barrier and innate immune function (45). It plays an important role in the epithelial transport of nutrients and metabolites and in the defense of penetrating microbiota (46). Intestinal barrier function depends primarily on the integrity of the epithelium and adjacent cell-to-cell connections (47). These connections are primary adhesion and tight adhesion complexes (48). A tight junction is usually between two adjacent cells at the top of the epithelium, where the plasma membrane is almost fused and tightly joined. Tight junctions, located at the top of intestinal epithelial cells, are the most important type of junctions between cells (49). When tight junctions are lost, intercellular permeability increases significantly, resulting in disruption of the intestinal epithelial barrier (50). *ZO-1*, *ZO-2*, and *Occludin* are classic tight junction proteins and can be used as common indicators to observe tight junction damage (51).

Smooth muscle contractions occur in two main ways. The calcium-dependent pathway mainly involves Ca^2+^ binding to calmodulin (*CaM*) to form a Ca^2+^-CaM complex (52). It can activate myosin light chain kinase (*MLCK*) in the cytoplasm. The non-calcium-dependent pathway is associated with *RhoA/Rho* kinase (*ROCK*) signaling pathway (53). The key signaling molecules of the *RhoA/ROCK* pathway include *RhoA*, myosin light chain kinase (*MLCK*), and myosin light chain (*MLC*) (54). Many studies have confirmed that gut microbiota is closely related to gastrointestinal dynamics, and a healthy gut microecology helps promote intestinal motility (55). The imbalance of intestinal flora may be the key factor in intestinal barrier dysfunction and intestinal motility dysfunction.

In this study, we sequenced the intestinal microflora of the mouse model. This study investigated the effect of selenium deficiency on intestinal epithelial cell tight junction, intestinal inflammation, and intestinal smooth muscle contraction due to changes in the intestinal flora. It provides theoretical guidance for the follow-up research.

## 2 Materials and Methods

### 2.1 Animals and Group

Female c57 mice aged 4-5 weeks (n = 40,18g body weight) were selected. These mice were divided randomly into two groups and 20 mice in each group. The environmental parameters such as temperature, relative humidity, wind speed, and the illumination of each group were controlled and kept consistent. Low selenium group (LG): 20 mice were fed with Se-deficient mouse diet (Se content: 0.01mg Se/kg). Normal Se group (NG): 20 mice were fed with normal Se content mouse diet (Se content: 0.15mg Se/kg). Rat food is customized for selenium deficiency rat food and normal rat food from Trophic Animal Feed High-tech Company, China. The mice were free to eat and drink. The mice died of cervical dislocation after being fed at room temperature for 50 days and were quickly sampled and stored in a -80°C refrigerator. The animal study was reviewed and approved by the Institutional Animal Care and Use Committee (IACUC) of Jilin University.

### 2.2 Histopathology Staining

Small intestinal tissue was fixed in 4% paraformaldehyde and paraffin-embedded overnight. The small intestine sections were cut into two μm thick and soaked overnight in ZGSJ (Masson A). The small intestine sections were stained with Weigert’s hematoxylin (Masson A and Masson B in equal amounts) for 1 minute, differentiated by 1% acid ethanol, Then it was stained in scarlet magenta solution (Masson D) for 6 min, differentiated in phospho-molybdenum-phosphotungstate solution (Masson E) for 1 min, and transferred directly (without rinsing) to aniline blue solution (Masson F) for 2-30s. Then, rinse briefly in distilled water for 2-5 minutes. Finally, dehydration through anhydrous ethanol, xylene transparent, and neutral sealant. The sections were scanned by Pannoramic 250 slide scanner (3D HISTECH). Micrographs were analyzed by the blind method. Collagen volume fraction (CVF) was observed by image analysis system software (HALO, Indica Labs, American). Technical support was provided by Servicebio, Inc. (Wuhan, China).

### 2.3 Evaluation of the Degree of Apoptosis in Late Apoptotic Cells

Chromosomal DNA double-strand or single-strand breaks produce sticky 3’-OH ends. Under the catalysis of deoxyribonucleotide terminal transferase (DTT), dUTP with fluorescein molecule is labeled to the 3’end of DNA. Observe the stained apoptotic cells through a fluorescence microscope. Dewax the paraffin sections to water, break the membranes, incubate the Tunel reaction solution, microwave repair, incubate the primary antibody overnight at 4°C, add the secondary antibody, DAPI staining the nucleus, anti-fluorescence quenching and mounting, microscopic examination and taking pictures, and observe the results.

### 2.4 Real-Time PCR

Total RNA was extracted from frozen intestinal tissue. The concentration and purity of RNA solution were determined by UV spectrophotometry at 260nm and 280nm. Targeting specific primers were synthesized by reverse transcription design of glyceraldehyde 3-phosphate dehydrogenase (GAPDH) based on known sequences. The ABI PRISM7700 processing system was used for real-time quantitative PCR. For each gene to be measured, the cDNA template and sample cDNA of the expressed gene is selected for PCR reaction. There are 40 cycles, such as 94°C for 30 seconds, 94°C for 5 seconds, and 60°C for 30 seconds. Each experiment was repeated three times, and each sample was repeated three times. GAPDH was used as an endogenous internal standard control. The primers were purchased from Sangon Biotech, Shanghai. Primer sequences are shown in **Table 1**.

**Table 1:** Primer sequence list.

IL-1β	Forward: 5′- TTCCCA TTAGACAACTGC-3′	BcL-w	Forward:5′-CCGTCTTGTGGCATTCTT-3′
	Reverse: 5′- CTGTAGTGTTGTATGTGATC -3′		Reverse: 5′-AGCACTGTCCTCACTGAT-3′
IL-6	Forward: 5′- CAGAACCGCAGTGAAGAG -3′	Pum	Forward:5′-GATTCGGAAGCAGCAGTT-3′
	Reverse: 5′- CAGAACCGCAGTGAAGAG -3′		Reverse: 5′-GGAGCAGCAGAGATGTATC-3′
TNF-α	Forward:5′- CTCA TTCCTGCTTGTGGC -3′	p53	Forward:5′-TGGAAGACAGGCAGACTT-3′
	Reverse: 5′- CACTTGGTGGTTTGCTACG -3′		Reverse: 5′-GTGATGATGGTAAGGATAGGT-3′
IL-10	Forward: 5′- CAGAGCCAAAGCAGTGAGC -3′	Bak	Forward:5′-GGAATGCCTACGAACTCTT-3′
	Reverse: 5′- TGACCCAGTCCATCCAGAG -3′		Reverse: 5′-CCAACAGAACCACACCAA-3′
NF-κB	Forward:5′-CCATAGCCATAGTTGCGGTCCTTC-3′	Caspase3	Forward:5′-TGGTTGACGCAGTAGAGA-3′
	Reverse: 5′- CGTTCTTCCCTCCCTTTTCCTTTCC-3′		Reverse: 5′-GACGCCTTCACACTTCAT-3′
IκB-α	Forward:5′-GAATCACCAGAACATCGTGAAG-3′	RIP1	Forward:5′-TGGTTGACGCAGTAGAGA-3′
	Reverse: 5′- CAGTACTCCATGATTAGCACCT-3′		Reverse: 5′-GACGCCTTCACACTTCAT-3′
p38	Forward:5′-GCCGCTTAGTCACATACC-3′	RIPK3	Forward:5′-TGCCTTGACCTACTGATTG-3′
	Reverse: 5′-GCCGCTTAGTCACATACC-3′		Reverse: 5′-TTCCGTGACATAACTTGACA-3′
ATG7	Forward:5′-GTGTACGATCCCTGTAACCTAG-3′	ZO-1	Forward:5′- AACCCGAAACTGATGCTGTGGATAG -3′
	Reverse: 5′- GATGCTATGTGTCACGTCTCTA-3′		Reverse:5′- CGCCCTTGGAATGTATGTGGAGAG -3′
ATG5	Forward:5′-AGTCAAGTGATCAACGAAATGC-3′	ZO-2	Forward:5′- CATGTCTCTAACGGATGCTCGGAAG -3′
	Reverse: 5′- TATTCCATGAGTTTCCGGTTGA-3′		Reverse:5′- GTTTAGGGCTGGGATGTTGATGAGG -3′
p62	Forward:5′-GAACACAGCAAGCTCATCTTTC-3′	Occludin	Forward:5′- TGGAGGCTATGGCTATGGCTATGG-3′
	Reverse: 5′- AAAGTGTCCATGTTTCAGCTTC-3′		Reverse:5′- TTACTAAGGAAGCGATGAAGCAGAAGG-3′
Beclin	Forward:5′-TAATAGCTTCACTCTGATCGGG-3′	E-cadherin	Forward: 5′- ACCAGCAGTTCGTTGTCGTCAC-3′
	Reverse: 5′- CAAACAGCGTTTGTAGTTCTGA-3′		Reverse: 5′- GTTCCTCGTTCTCCACTCTCACATG-3′
LC3α	Forward:5′-CTGTCCTGGATAAGACCAAGTT-3′	CaM	Forward:5′-ACAAGGATGGGAATGGTTACAT-3′
	Reverse: 5′- GTCTTCATCCTTCTCCTGTTCA-3′		Reverse: 5′- TGCAGTCATCATCTGTACGAAT-3′
PERK	Forward:5′-GTAGCCACGACCTTCATC-3′	MLC	Forward:5′-GATAGCCATCAGCAGCCTCACATC-3′
	Reverse: 5′-GTAGCCACGACCTTCATC-3′		Reverse: 5′- GCAACAGGAGCAGCAGGAGAAC-3′
IRE1	Forward:5′-TTGAAGTGGACAGTGAAGG-3′	MLCK	Forward:5′-GGGCTGCCTCTCATCATCAATACG-3′
	Reverse: 5′-TTGAAGTGGACAGTGAAGG-3′		Reverse: 5′- TGGATTCTGCTTCTGTGGGTAGGG-3′
elF2α	Forward:5′-TGGTGGTTATCCGTGTTG-3′	RhoA	Forward:5′-ACGGTGTTTGAAAACTATGTGG-3′
	Reverse: 5′-CCGATTGCTTGAAGATGTC-3′		Reverse: 5′- GACAGAAATGCTTGACTTCTGG-3′
GRP78	Forward:5′-GTCAGGGAGAGGAGGAAT-3′	Rho	Forward:5′-GTCTGATCTTCGTGGTAGACTG-3′
	Reverse: 5′-TGGTGTCACTTATGGTAGAA-3′		Reverse: 5′- CTCATCTCCCGGTCATTGATAA-3′
CHOP2	Forward:5′-TACACCACCACACCTGAA-3′	GAPDH	Forward: 5′-CCCAGAAGACTGTGGATGG-3′
	Reverse: 5′-GCACCACTACACCTGATAG-3′		Reverse: 5′- ACACATTGGGGGTAGGAACA-3′
BcL-2	Forward:5′-CCTCCAATACTCACTCTGTC-3′		
	Reverse: 5′-TACCTGCGTTCTCCTCTC-3′		

### 2.5 Intestinal Flora Assay

In this study, genomic DNA was extracted from intestinal contents by CTAB or SDS. After that, agarose gel electrophoresis was used to detect the purity and concentration of DNA. An appropriate amount of DNA was taken into the centrifuge tube and diluted to 1ng/μ L with sterile water. Using diluted genomic DNA as a template, specific primers with barcodes were used according to the 16S V3-V4 region of the sequencing area. New England Biolabs Phusion^®^ High Fidelity PCR Master Mix with GC Buffer and High Fidelity enzyme for PCR amplification efficiency and accuracy. PCR products were detected by electrophoresis with 2% agarose gel. The qualified PCR products were purified by magnetic beads, quantified by enzyme standard, and mixed in equal quantities according to the concentration of PCR products. After full mixing, PCR products were detected by 2% agarose gel electrophoresis, and the target bands were recovered using gel recovery kits provided by Qiagen. TruSeq^®^ DNA PCR-free Sample Preparation Kit was used for library construction. The constructed library was quantified by Qubit and Q-PCR. After the library was qualified, NovaSeq6000 was used for machine sequencing. Each sample data was separated from the off-machine data according to the Barcode sequence and PCR primer sequence. Qiime software (Version 1.9.1) was used to calculate and analyze the data. This experiment was commissioned by Novogene Tianjin Company.

### 2.6 Statistical Analysis

SPSS 22.0 statistical software was used for statistical analysis. All data were expressed as ± standard deviation (SD) measurements. The data were compared by the T-test method. The significance of the difference criterion is P<0.05.

## 3 Result

### 3.1 Se Deficiency Resulted in Changes in the Composition and Structure of Intestinal Flora in Mice

#### 3.1.1 Species Lightning Strike Curve

**Figure 1:**
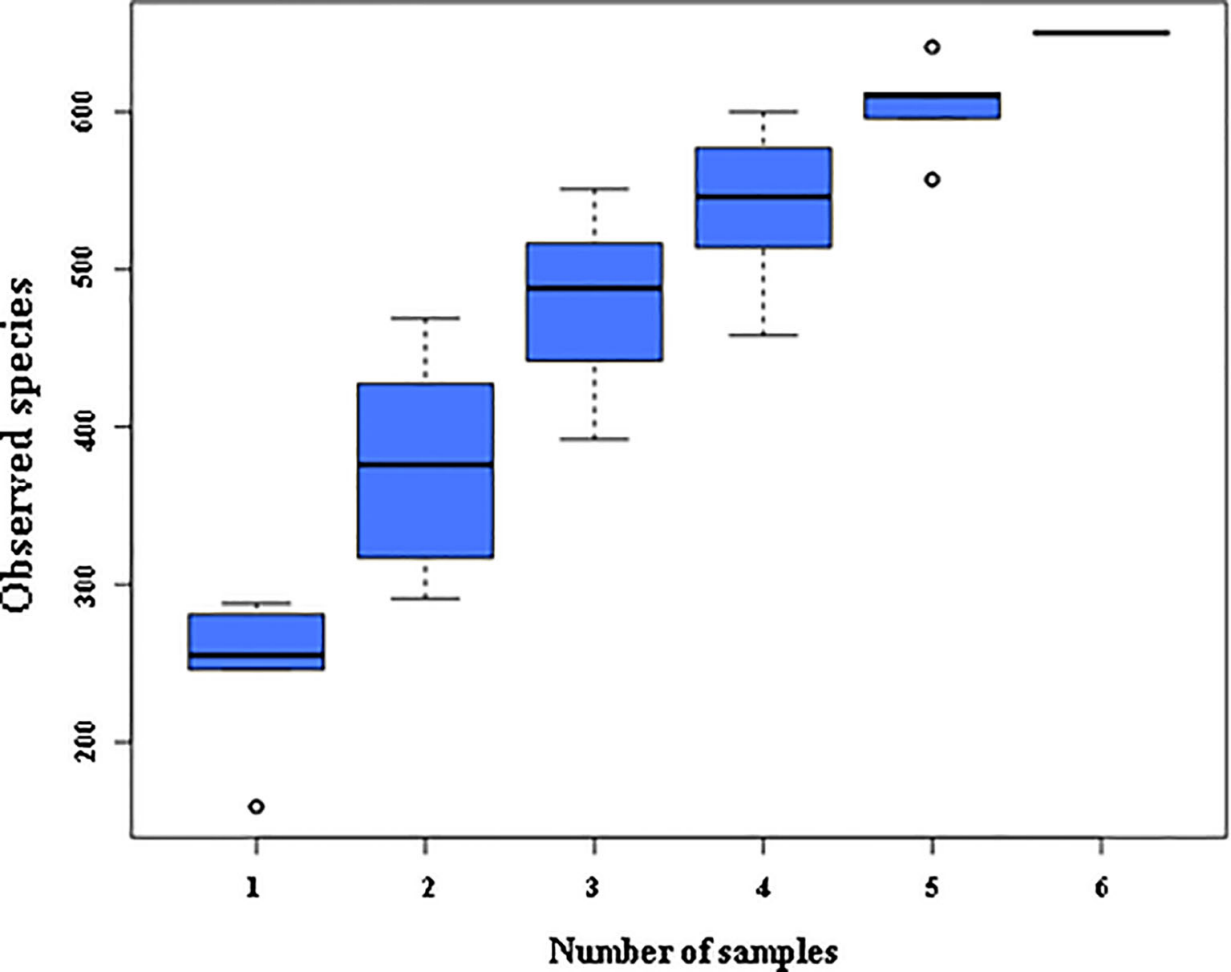
Species accumulation box plot. The abscissa is the sample size; The y-coordinate is the number of OTU after sampling.

The species accumulation curve is used to determine whether the available sample size is sufficient for a high-throughput community analysis of the species. If the upward trend of this curve is always steep, it indicates that the sample size is too small to support the following analysis. As the number of samples increases, the upward trend of the curve is relatively gentle, indicating that the original sample size is large. Therefore, it is necessary to measure the cumulative curve of species before all kinds of analyses of fecal flora. If the cumulative curve always shows an upward trend, it indicates that the sample size is insufficient to support the analysis of species composition structure. On the contrary, if the curve rises gently with the increase of samples, it indicates that the sample size is sufficient to reflect the species composition of the community. It can be seen in **Figure 1** that the species accumulation curve of mouse intestinal contents collected in this experiment rises gently, indicating that the collected feces samples are sufficient for species composition analysis in this experiment.

### 3.2 Analysis of Relative Abundance of Species

**Figure 2:**
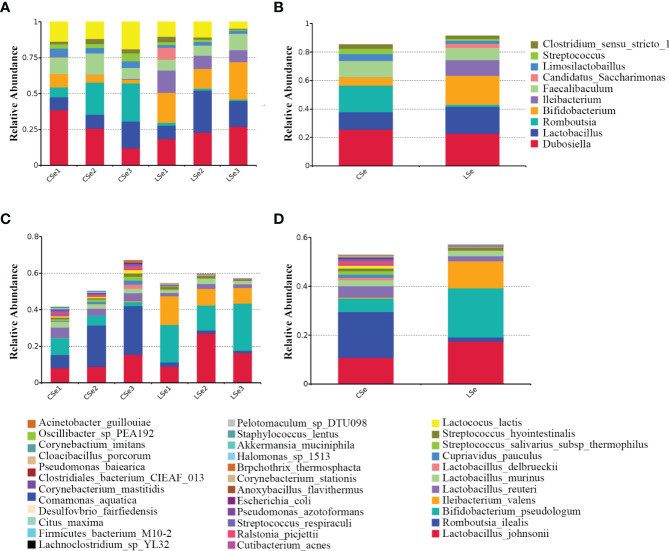
Histogram of relative abundance of species. **(A)** Histogram of relative abundance of sample species (Top 10, Genus). **(B)** Histogram of relative abundance of grouped species (Top 10, Genus). **(C)** Histogram of relative abundance of sample species (Top 35, Species). **(D)** Histogram of relative abundance of grouped species (Top 35, Species).

**Figures 2A, B** is a species composition analysis of all samples at the genus level. When analyzed by group, it could be seen that the composition of fecal flora changed significantly after the LSe group. The most abundant strains in the CSe were Dubosiella (25.4976%), Lactobacillus (12.2355%), and Romboutsia (18.7987%) Faecalibaculum (11.1052%). The bacteria with the highest abundance in LSe group were Dubosiella (22.8274%), Lactobacillus (18.7323%), Bifidobacterium (20.2125%) and Ileibacterium (11.0841%). The abundance of Lactobacillus, Bifidobacterium, and Ileibacterium in the LSe group was higher than in the CSe. Romboutsia abundance in the LSe group was significantly lower than that in CSe. **Figures 2C, D** shows the species composition analysis of all samples at the species level. The abundance of Lactobacillus_johnsonii, Bifidobacterium_pseudolongum, and Ileibacterium_valens in the LSe group was significantly higher than that in the CSe group. The abundance of all other bacteria was lower or none in the CSe group.

### 3.3 Phylogenetic Tree Analysis of Genus Level Species

**Figure 3:**
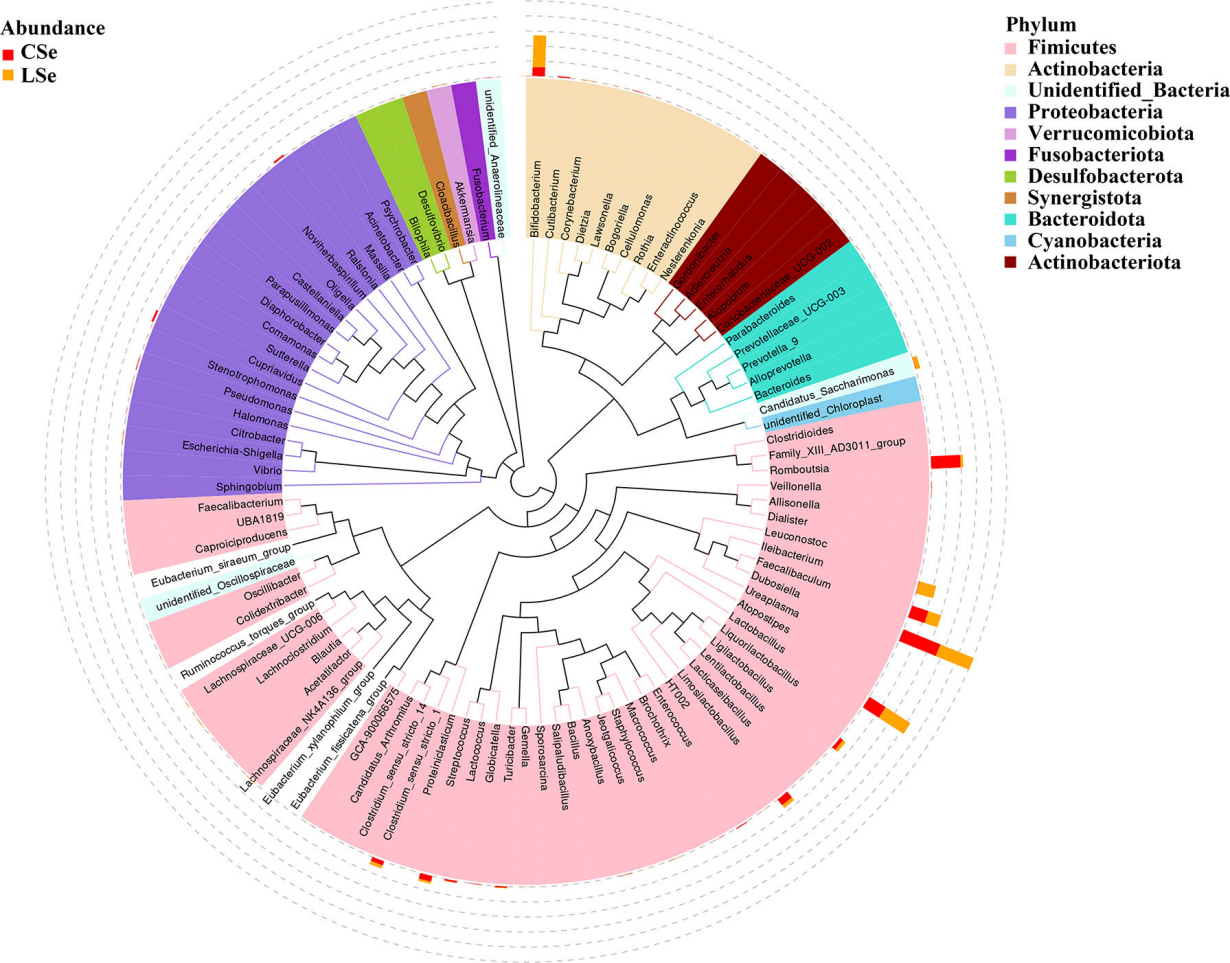
Phylogenetic tree analysis of genus level species.

The representative sequences of the top100 genera were obtained by multiple sequences alignment to further study the phylogenetic relationships of genus-level species. The resulting display was shown in **Figure 3**. The phylogenetic tree was constructed by the representative sequences of the horizontal species of the genus. The colors of branches and fan-shaped segments represented the corresponding phylum, and the accumulation histogram outside the fan ring represented the abundance distribution information of the genus in different samples. The relative dominant genus and phylum of LSe could be identified. Firmicutes phylum Ileibacterium is the most obvious bacterium.

### 3.4 Alpha Diversity Index Analysis

#### 3.4.1 Analysis of Differences in Alpha Diversity Index Between Groups

**Figure 4:**
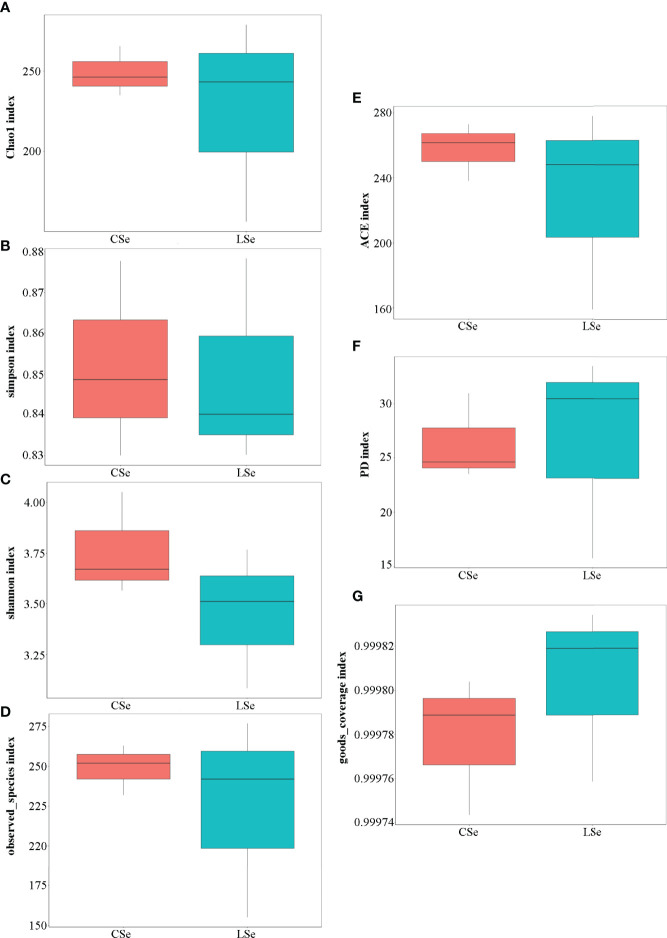
Alpha Diversity index analysis. **(A)** Chao1 index; **(B)** Simpson index; **(C)** Shannon index; **(D)** Observed species index; **(E)** ACE index; **(F)** PD index **(G)** Good’s Coverage index.

**Figure 4** shows the analysis results of Alpha Diversity index. The repeatability among three samples was poor in the LSe group. Chao1 index reflects the presence of low abundance species, and the CSe group is higher than the LSe group. The species richness of the LSe group was lower than that of the CSe group. Simpson index and Shannon index indicated that the diversity and evenness of species distribution of the CSe group were better than that of the LSe group. ACE index and Observed species index reflect the number of randomly selected bacterial community species in the CSe group is more than that in the LSe group. PD index indicated that species in the LSe group had more complex genetic relationships and longer evolutionary distances. Good’s Coverage index reflects the sequencing depth.

#### 3.4.2 Analysis of species diversity curve

**Figure 5:**
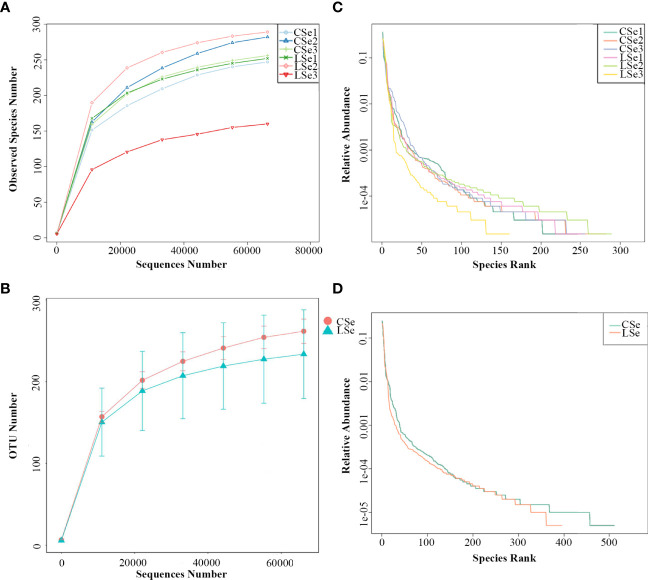
Species dilution curve. **(A)** rarefaction Curve (Simple); **(B)** Rank Abundance (Simple);(C) rarefaction Curve (Group); **(D)** Rank Abundance (Group).

The rarefaction Curve and Rank Abundance curve are usually describing the diversity of samples within groups. In **Figures 5A, B**). The dilution curve reflects the rationality of the two groups of sequencing data, and indirectly reflects the richness of species in the samples. When the curve tends to be flat, the sequencing data amount is progressive and reasonable, and more data amount will only produce a few new species. The rank Abundance curve in **Figures 5C, D** can intuitively reflect the Abundance and evenness of species in samples. The greater the span of the curve along the horizontal axis, the higher the species richness. In the vertical direction, the smoothness of the curve reflects the uniformity of species in the sample. Vertically, the smoothness of the curve reflects the uniformity of species in the sample. The flatter the curve, the more evenly distributed the species. In general, the species richness and evenness of the CSe group were better than that of the LSe group.

### 3.5 Analysis of Differences in Beta Diversity Index Between Groups

#### 3.5.1 Beta Diversity Analysis

**Figure 6:**
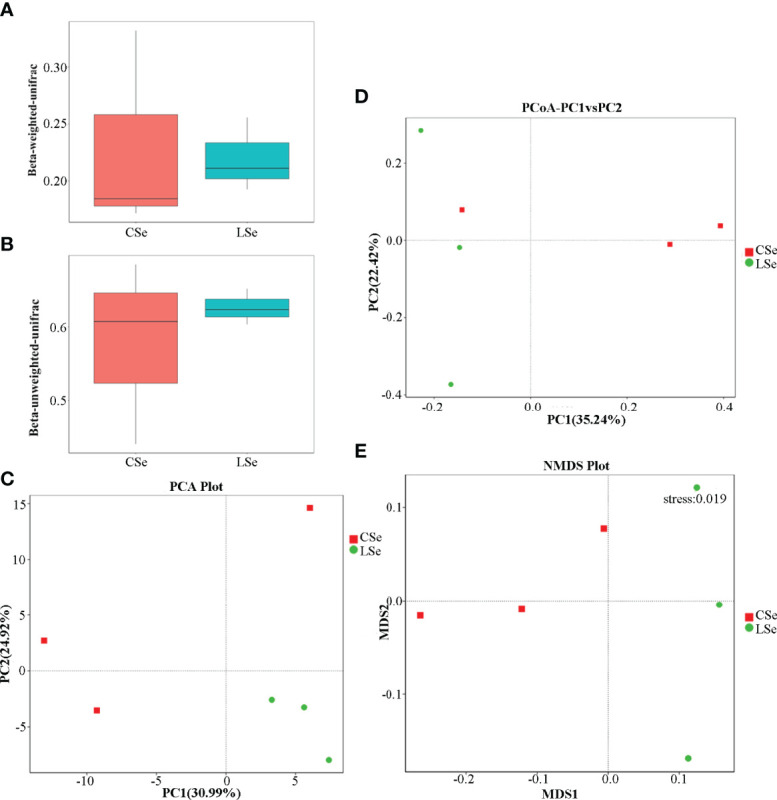
Analysis of differences in Beta diversity index between groups. **(A)** Beta-weighted-unifrac index; **(B)** Beta-unweighted-unifrac index; **(C)** PCA (Principal Component Analysis); **(D)** PCoA (principal coordinate analysis); **(E)** NMDS (non-metric multi-dimensional Scaling).

Beta Diversity is a comparative analysis of microbial community composition of different samples. As shown in **Figures 6A, B**, Unifrac distance is a method to calculate the distance between samples by using the evolutionary information between microbial sequences in each group. Weighted Unifrac distance and Unweighted Unifrac distance were used to measure species diversity differences between the two samples. The results showed that the diversity difference between the CSe group was smaller than that of the LSe group.

#### 3.5.2 Principal Component Analysis

PCA (Principal Component Analysis) is a method to extract the most important elements and structures in data based on Euclidean Distances. PCA analysis can extract two coordinate axes that reflect the difference between samples to the greatest extent. The differences in multi-dimensional data are reflected in the two-dimensional coordinate graph, and the simple rules under the complex data background are revealed. The more similar the community composition of the samples is, the closer they are to the PCA diagram. As shown in **Figure 6C**, at the genus level, the community composition of the LSe group samples was more similar.

#### 3.5.3 Principal Coordinate Analysis

To characterize the overall differences in the gut microbiome between groups, we performed a principal coordinate analysis (PCoA) of unweighted UniFrac distances. There were significant differences between the LSe group and CG (**Figure 6D**). The results also showed that the bacterial community structure in the feces of mice was significantly changed by Se deficiency.

#### 3.5.4 Non-Metric Multi-Dimensional Scaling (NMDS)

NMDS (non-metric multi-dimensional Scaling) is a ranking method suitable for ecological studies. NMDS is a nonlinear model based on bray-Curtis distance, which is reflected in a two-dimensional plane in the form of points according to the species information contained in the sample. It can better reflect the nonlinear structure of ecological data. NMDS analysis can reflect the differences between and within groups of samples. NMDS analysis results based on OTU level are shown in **Figure 6E**. There are significant differences between the two groups of samples.

### 3.6 LefSe Analysis of Bacterial Community Structure in Small Intestinal Contents of Mice

**Figure 7:**
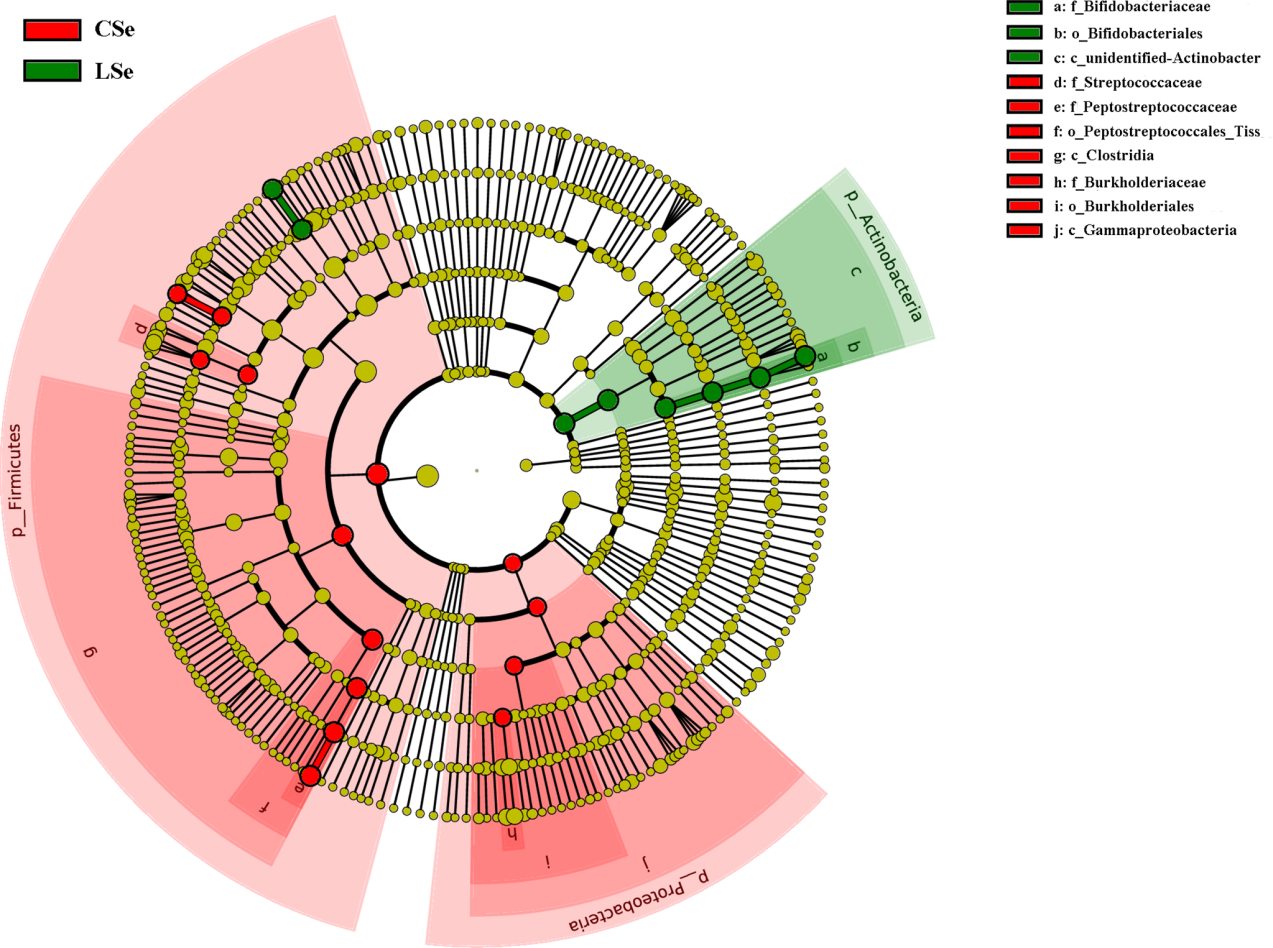
LefSe analysis of bacterial community structure in small intestinal contents of mice.

LEfSe is used to detect OTUs or bacterial system types (phylum to order level) in different feeding groups. In **Figure 7**, Actinobacteria and Ileibacterium_valens in the LSe group were significantly higher than those in the CG group, which might be the reason for the change in intestinal mucosal barrier in mice.

### 3.7 Tax4Fun Function Predictive Analysis

**Figure 8:**
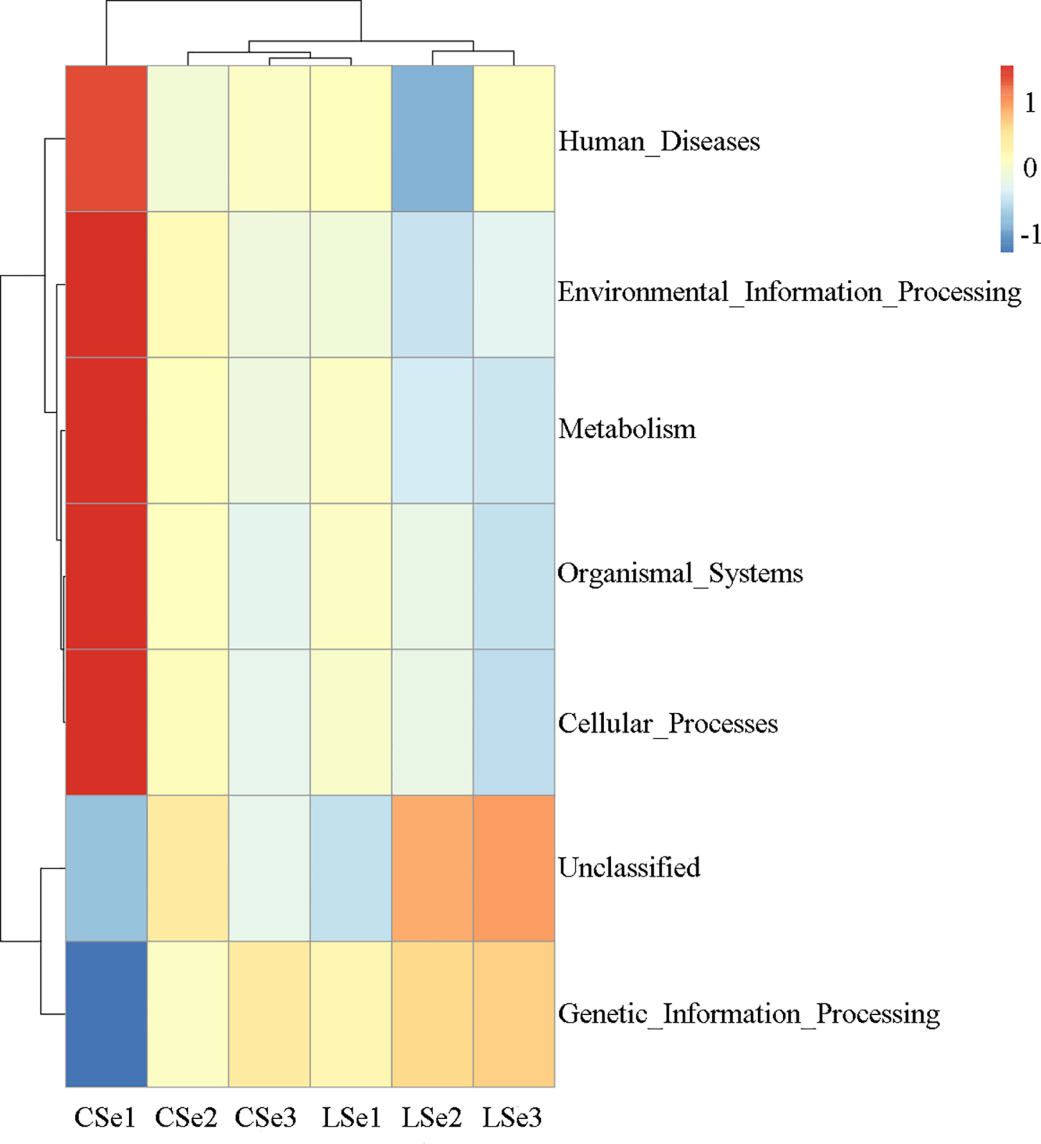
Tax4Fun function predictive analysis. According to the functional notes and abundance information of samples in the database, the top 35 functions and their abundance information in each sample were selected to draw a heat map. And cluster from the level of functional difference. The abscissa is the sample name and the ordinate is the feature comment.

Tax4Fun function prediction is used by the nearest neighbor method based on the minimum 16SrRNA sequence similarity. The specific method was to extract the 16S rRNA gene sequence of the whole genome of prokaryotes from the KEGG database and compare it with the SILVA SSU Ref NR database (BLAST bitscore >1500) using the BLASTN algorithm to establish a correlation matrix. The whole-genome functional information of prokaryotes annotated by UProC and PAUDA in the KEGG database was corresponding to the SILVA database to realize the functional annotation of the SILVA database. SILVA database sequence was used as a reference sequence to cluster OTU and obtain functional annotation information. Changes of Intestinal microflora induced by Selenium Deficiency and their relationship with Human Diseases, Environmental Information Processing, Metabolism, Organismal Systems, Cellular Processes Genetic information processing. The result of the annotation is shown in **Figure 8**.

### 3.8 Se Deficiency Caused Apoptosis and Injury of Small Intestine in Mice

**Figure 9:**
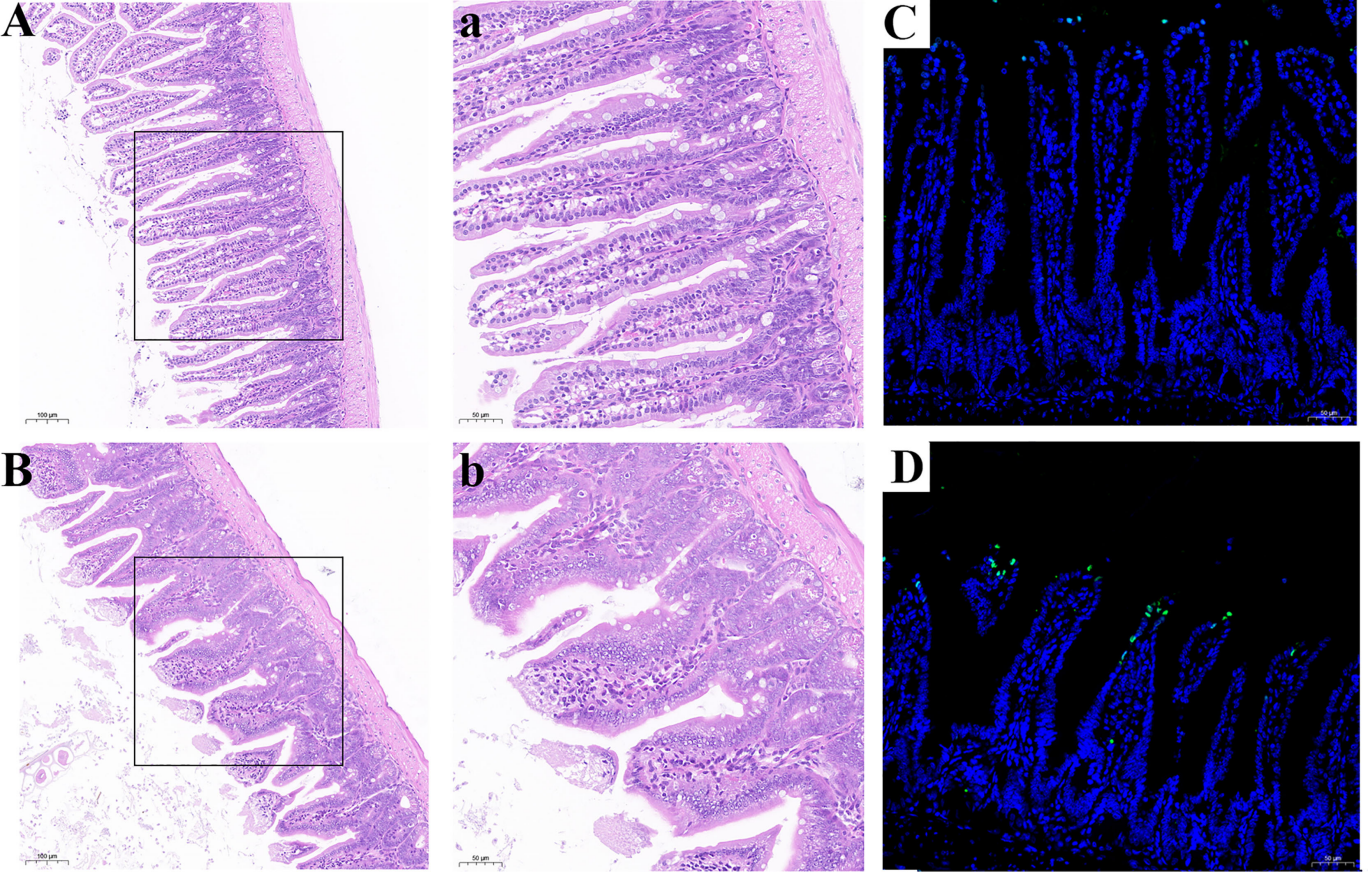
Se deficiency caused apoptosis and injury of small intestine in mice. **(A, B)** Histopathology of small intestine. **(A)** CSe group; (a) An enlarged image of CSe Group; **(B)** LSe group; (b) An enlarged image of LSe group; **(C, D)** TUNEL analysis of small intestinal tissue; **(C)** CSe group; **(D)** LSe group.

Histological analysis was shown in **Figures 9A, B**. Compared with the CSe group, the LSe group had lower villi height and even fractures. Goblet cells are reduced. The mucosa and muscular arrange the cells lose. TUNEL test results are shown in **Figures 9 C, D**, and the green fluorescence intensity of the LSe group is significantly higher than that of the CSe group. It suggests that selenium deficiency promotes apoptosis of small intestinal cells.

### 3.9 Selenium Deficiency Leads to Inflammation, Autophagy, Endoplasmic Reticulum Stress, Apoptosis, Tight Junctions and Smooth Muscle Contraction in Small Intestinal Tissues of Mice

**Figure 10:**
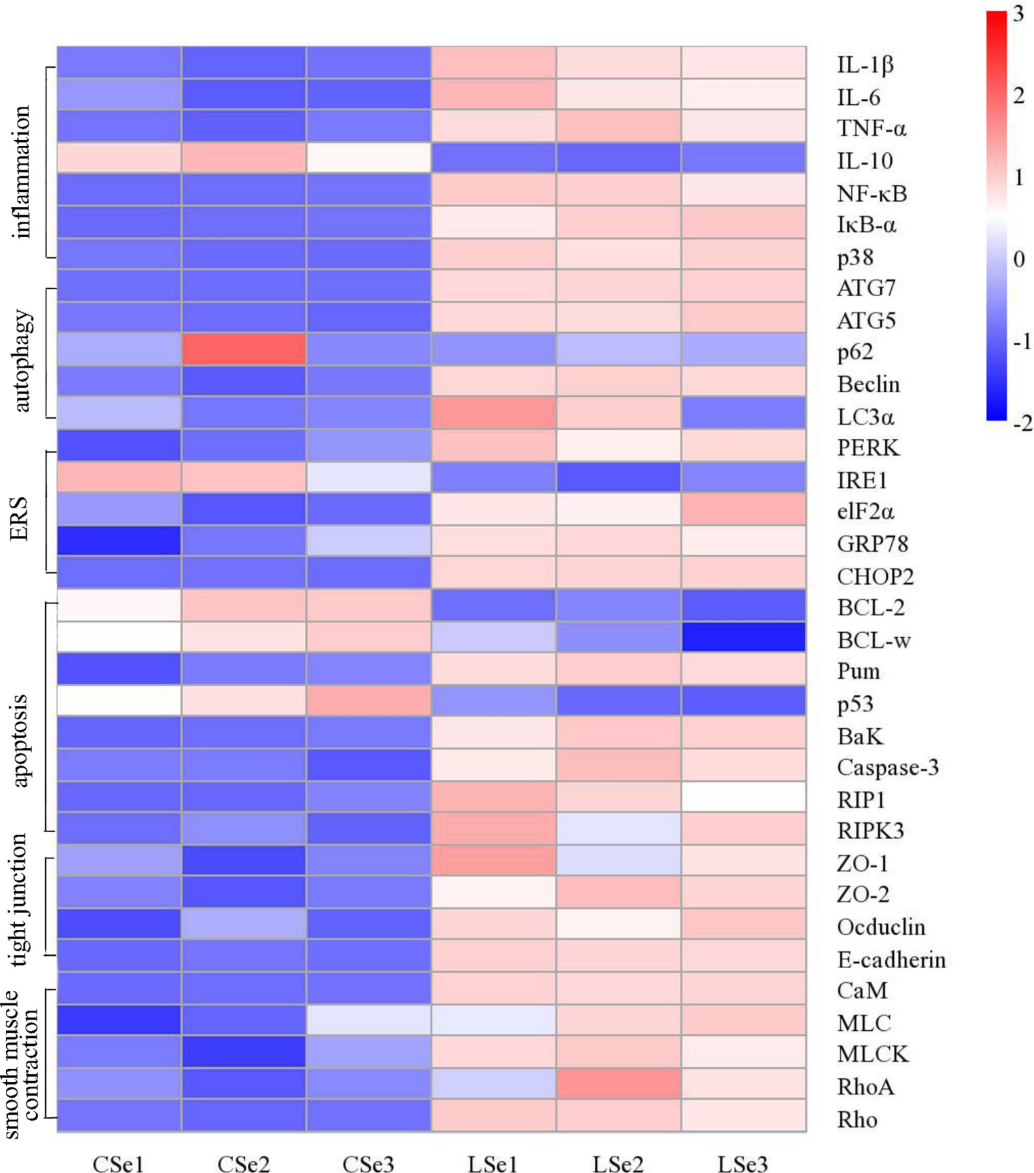
Analysis of mRNA expression level in small intestine tissue. The Ct values measured by PCR were calculated by 2-△△ Ct and then standardized. The abscissa is the sample name and the ordinate is the gene name. p < 0.05.

We examined gene levels in the small intestine related to inflammation, autophagy, endoplasmic reticulum stress, apoptosis, tight junctions and smooth muscle contraction. The results were shown in **Figure 10**. The mRNA levels of *NF-κB, IκBα*, *p38, IL-1β*, and *TNF-α* in the LSe group were significantly increased, while the mRNA levels of *IL-10* were significantly decreased. It is evidence of inflammation in the small intestine. The mRNA levels of *Beclin, ATG7, ATG5*, and *LC3α* were significantly increased in the LSe group, while the mRNA levels of *p62* were slightly decreased compared with the CSe group. It indicates that the cells of the small intestine are autophagy. Compared with CSe, mRNA levels of *PERK, IRE1, elF2α*, *GRP78*, and *CHOP2* were significantly increased. It indicates endoplasmic reticulum stress in the cells of the small intestine. The mRNA levels of *BaK*, *Pum*, *Caspase-3*, *RIP1*, and *RIPK3* were significantly increased in the LSe group, while the mRNA levels of *BcL-2* and *BcL-w* were significantly decreased in the LSe group compared with the CSe group. It indicates that the cells of the small intestine have undergone apoptosis. The mRNA levels of tight junction proteins such as *ZO-1, ZO-2, Occludin*, and *E-cadherin* were significantly increased. It suggests that the tight junctions of the small intestine were broken in the LSe group. The expression of LSe smooth muscle contraction-related genes such as *CaM*, *MLC*, *MLCK*, *Rho*, and *RhoA* increased. It indicates abnormal contraction of the small intestine smooth muscle.

## 4 Discussion

Se is a micronutrient. It has biological functions such as anti-oxidation, anti-cancer, and enhancing immunity (2, 3). Se deficiency can cause weakened immunity, muscle atrophy, arrhythmia, and other symptoms. Previous studies have shown that Se regulates gut microbiota. Some studies have also found that chronic Se deficiency can cause chronic diarrhea (56). Our study shows that Se deficiency does cause intestinal inflammation in mice. It confirms this view. What is the cause of Se deficiency leading to diarrhea? Is it related to changes in gut flora?

Intestinal flora has become a new hot research field. The interaction between intestinal flora and Se in food mainly focuses on the composition of symbiotic flora, the regulation of the metabolic process, and the function of the intestinal mucosal barrier. To further evaluate the effects of Se deficiency on intestinal microbiota in mice, we extracted the contents of the small intestine for 16S high-throughput sequencing analysis. The results showed that the intestinal microbiota of the LSe group changed significantly compared with that of CG. The strains with the highest abundance in the CG were Dubosiella, Romboutsia, Lactobacillus, and Faecalibaculum. Dubosiella, Bifidobacterium, Lactobacillus, and Ileibacterium had the highest abundance in the LSe group. The composition of microflora changed obviously. Compared with CG, the abundance of Lactobacillus, Bifidobacterium, and Ileibacterium with LSe significantly increased, while the abundance of Romboutsia significantly decreased or almost disappeared. The abundance changes of Ileibacterium and Romboutsia are the most obvious. The abundance changes of these two bacteria likely cause the abundance changes of Dubosiella and Lactobacillus. But this still needs to be further studied. The result suggests that selenium deficiency can lead to changes in the composition of intestinal flora.

Our study showed significant pathological changes in small intestinal tissues of mice in the LSe group compared with CSe. The studies on gene levels showed that cytokines *IL-1β, TNF-α*, and *IL-6* were significantly elevated. The LSe group also significantly increased the expression of *NF-κB* and the *IκBα* gene in the classic inflammatory signaling pathway. This confirmed that selenium deficiency can cause inflammation of the small intestine through intestinal flora.

Autophagy plays an important role in regulating cell death. *Beclin* is an autophagy-related gene and participates in the formation of autophagosomes (57). *ATG5* and *ATG7*, *LC3α* play a key role in autophagy elongation (58). *p62* is considered one of the markers of autophagy ability (59). The result was found that the mRNA levels of *Beclin*, *ATG5*, *ATG7*, and *LC3α* in the LSe group were significantly increased, while the mRNA levels of *p62* were significantly decreased. It proves that selenium deficiency promotes autophagy through intestinal flora.

ER is one of the most important organelles in the cell, which is mainly involved in protein processing and modification, guiding its correct folding and assembly. ERS may be affected by various stimuli such as infection and oxidative stress. *PERK*, *IRE1*, *elF2α*, *GRP78*, and *CHOP2* are key genes in er stress (60). PCR analysis showed that the mRNA levels of *PERK*, *IRE1*, *elF2α*, *GRP78*, and *CHOP2* were increased in the LSe group. Other studies have shown that dysbiosis can cause er stress. This proves that selenium deficiency promotes endoplasmic reticulum stress through intestinal flora.

TUNEL analysis showed that the apoptosis level of LSe intestinal epithelial cells was increased. *BaK* induces apoptosis through mitochondrial pathway activation of *Caspase-3* (61). p53 can promote cell apoptosis by blocking cell cycle activation of *Caspase-3* (62). *Pum* gene can promote cell apoptosis, *BcL-2* and *BcL-w* have an anti-apoptotic effect (63). *RIP1* and *RIPK3* play an important role in necrotic apoptosis (64). It was confirmed that the mRNA levels of *BaK*, *Pum*, *Caspase-3*, *RIP1*, and *RIPK3* were significantly increased in the LSe group, while the mRNA levels of *BcL-2* and *BcL-w* were significantly decreased in the LSe group compared with the CSe group. It indicates that the cells of the small intestine have undergone apoptosis. Selenium deficiency induces apoptosis of intestinal epithelial cells through intestinal flora.

Tight junction is an important structure to maintain a balance of intestinal mucosal barrier function. *ZO-1*, *ZO-2*, *Occludin*, and e-cadherin are important components of tight junction. The mRNA levels of tight junction proteins *ZO-1*, *ZO-2*, *Occludin*, and *E-cadherin* were significantly increased in the LSe group. The result suggests that the tight junctions of the small intestine were broken in the LSe group. It Indicates that selenium deficiency can disrupt the intestinal flora by tightly connecting them.

Abnormal contraction of intestinal smooth muscle can cause diarrhea with intestinal motility disorder. The expression of smooth muscle contractile related genes such as *CaM*, *MLC*, *MLCK*, *Rho* and *RhoA* increased. The result confirmed abnormal contraction of small intestinal smooth muscle in the LSe group. Selenium deficiency can cause abnormal contraction of intestinal smooth muscle through the intestinal flora.

In general, the changes in the abundance of Lactobacillus, Bifidobacterium, Ileibacterium and Romboutsia may be related to intestinal tissue cell inflammation, autophagy, endoplasmic reticulum stress, apoptosis, tight junction and abnormal smooth muscle contraction. Romboutsia is a bacterium that needs selenium to thrive properly. But the specific role each of these bacteria plays in selenium deficiency-induced intestinal inflammation is unknown. That remains to be explored.

In conclusion, long-term dietary Se deficiency does lead to changes in the composition of intestinal flora. Se deficiency can cause significant changes in species abundance of Lactobacillus, Bifidobacterium, Ileibacterium and Romboutsia. Species abundance of Lactobacillus, Bifidobacterium and Ileibacterium may be positively correlated with intestinal inflammation. Romboutsia was negatively correlated with intestinal inflammation. Intestinal microflora may play an important role in the response of intestinal tissue cell inflammation, autophagy, endoplasmic reticulum stress, apoptosis, tight junction, and abnormal smooth muscle contraction induced by selenium deficiency.

## Data Availability Statement

The original contributions presented in the study are included in the article/supplementary material. Further inquiries can be directed to the corresponding authors.

## Ethics Statement

The animal study was reviewed and approved by Institutional Animal Care and Use Committee (IACUC) of Jilin University.

## Author Contributions

FW, NS, and WZ conceived and designed the research; FW, NS, HZ, and YG performed the research and acquired the data, and FW analyzed and interpreted the data and wrote sections of the manuscript. All authors contributed to manuscript revision, read, and approved the submitted version.

## Conflict of Interest

The authors declare that the research was conducted in the absence of any commercial or financial relationships that could be construed as a potential conflict of interest.

## Publisher’s Note

All claims expressed in this article are solely those of the authors and do not necessarily represent those of their affiliated organizations, or those of the publisher, the editors and the reviewers. Any product that may be evaluated in this article, or claim that may be made by its manufacturer, is not guaranteed or endorsed by the publisher.
